# Ultrasound-Assisted Extraction Optimization and Cultivar Screening for Polyphenol Recovery from Thinned Peach Fruit: A Comprehensive Evaluation of 179 Cultivars

**DOI:** 10.3390/foods14111897

**Published:** 2025-05-27

**Authors:** Shenge Li, Jianlan Xu, Zhixiang Cai, Shaolei Guo, Mingliang Yu, Zhijun Shen

**Affiliations:** Jiangsu Key Laboratory of Horticultural Crop Genetic Improvement, Institute of Pomology, Jiangsu Academy of Agricultural Sciences, Nanjing 210014, China; lishenge@jaas.ac.cn (S.L.); jlxujaas1976@aliyun.com (J.X.); czx1y05@163.com (Z.C.); 20200041@jaas.ac.cn (S.G.); mly@jaas.ac.cn (M.Y.)

**Keywords:** thinned peach, polyphenols, antioxidation, ultrasonic-assisted extraction, resources

## Abstract

Thinned peach fruit represents a substantial source of polyphenols, primarily due to its early developmental stage. Utilizing ultrasound-assisted extraction optimized through a Box–Behnken design, we determined the optimal extraction parameters to be 45 min, 360 W, a liquid-to-solid ratio of 15:1 mL/g, and a temperature of 70 °C. Under these conditions, the total phenolic content (TPC) achieved was 1.12 g GAE/kg FW, with an extraction efficiency of 97.06%. Additionally, an extensive evaluation of 179 peach cultivars revealed that wild accessions possessed significantly higher polyphenol content, including TPC, total flavonoid content (TFC), and total anthocyanin content (TAC), alongside enhanced antioxidant activities as measured by ferric reducing antioxidant power (FRAP), 2,2′-Azino-di-3-ethylbenzthiazoline Sulfonic Acid (ABTS), and 2,2-Diphenyl-1-picrylhydrazyl (DPPH) radical scavenging assays, in comparison to landraces and cultivated varieties. Notably, the wild accession ‘Gansu Peach 2’ exhibited the highest TPC (2.61 g GAE/kg FW), whereas the landrace ‘Early White Blossom Peach’ demonstrated the highest TFC (137.32 g RTE/kg FW), TAC (25.30 g PAE/kg FW), and antioxidant capacity. Additionally, as expected, significant positive correlations (0.73 < *r* < 0.96) were also observed between polyphenol components and antioxidant activities (*p* < 0.0001). This study establishes a foundational framework for the utilization of thinned peach fruit as valuable polyphenol-rich resources.

## 1. Introduction

Peach trees demonstrate remarkably high fruit set rates, necessitating the removal of 30–50% of fruit 4–5 weeks post-bloom to ensure marketable fruit quality and sustainable orchard productivity [1,2]. In 2023, peach cultivation area expanded to 890,000 ha (FAO) in China, with principal production regions achieving yields of approximately 1500 kg/ha of thinned fruit, amounting to a national total of 1.33 million tons annually [3]. Paradoxically, less than 5% of this biomass is utilized as low-value animal feed, while more than 95% is discarded untreated in orchards [4]. This disposal strategy creates a dual sustainability crisis: (1) massive waste of bioactive-rich organic resources, and (2) ecological hazards from fungal pathogen proliferation (particularly *Monilinia* spp.) that elevate orchard disease pressure by 40–60% [5]. Addressing this challenge requires urgent development of valorization strategies that convert thinning byproducts into value-added products while mitigating ecological risks.

Peach thinning fruit constitutes a significant bioactive resource, characterized by a rich composition of polysaccharides, organic acids, amino acids, and polyphenols, each with unique compositional profiles. This study addresses two unresolved challenges in the valorization of these byproducts: (1) the absence of specific protocols for the efficient extraction of unstable phenolics from peach thinning byproducts, and (2) the critical need for systematic germplasm screening to identify cultivars with enhanced polyphenolic potential. Compared to mature fruit, the carbohydrate fraction of peach thinning fruit is predominantly composed of galactose, galacturonic acid, and arabinose, which have demonstrated prebiotic effects in alleviating gut dysbiosis and cellular dysfunction in mice with disrupted circadian rhythms [6,7]. Although the polyphenolic profile is compositionally similar to that of mature fruit, it exhibits concentrations that are 10 times higher, with neochlorogenic acid (NCGA), catechin, and chlorogenic acid (CGA) forming the core antioxidant system. These compounds provide antioxidant activities that are 1.3 to 11.2 times greater, as evidenced by FRAP, DPPH, and ABTS assays, and show strong positive correlations with polyphenolic content [8,9,10,11]. Specific phenolic compounds exhibit distinct bioactivities: CGA mitigates reactive oxygen species (ROS)-induced apoptosis of retinal ganglion cells in glaucoma [12], whereas NCGA provides neuroprotection via anti-inflammatory pathways in microglia [13]. Furthermore, notable cultivar-specific bioactivities have been identified: light-fleshed varieties, such as ‘Spring Time’ and ‘Madison’, which are rich in phenolic acids and flavonols, effectively inhibit α-amylase, a target for type 2 diabetes management. In contrast, carotenoid-rich yellow-fleshed cultivars, such as ‘Harrow Diamond’ and ‘Harrow Beauty’, demonstrate strong inhibition of pancreatic lipase, a target for obesity treatment [14]. Interestingly, the peach cultivars in this study represent over 80% of China’s commercial peach cultivars, ensuring the translational relevance of our findings. These findings will underscore the potential of peach thinning fruit as a valuable resource for the development of targeted bioactive compounds.

The optimization of ultrasound-assisted extraction (UAE) was prioritized due to its distinctive ability to address existing challenges [15]. Current methods for extracting polyphenols from peaches yield modest amounts (0.36–1.06 g GAE/kg FW) [16,17], and traditional thermal techniques result in significant anthocyanin degradation (32–45% loss) and require extended processing times (180 min). UAE’s mechanoacoustic effects facilitate: (1) enhanced disruption of cell walls through cavitation, (2) minimized thermal degradation via precise temperature regulation, and (3) improved extraction efficiency through intensified mass transfer. Preliminary experiments demonstrated UAE’s superiority, achieving yields 66% higher (1.06 vs. 0.36 g GAE/kg FW) in 78% less time (39 vs. 180 min) compared to aqueous extraction [17]. Despite the high polyphenol content in peach thinning fruit, extraction is hindered by factors such as complex bound or polymerized forms, physicochemical instability, and technological limitations in purification. This study systematically optimizes UAE parameters, including solvent concentration, power, duration, and temperature, to develop a robust extraction protocol. This approach addresses critical barriers to industrial application and facilitates the high-value utilization of these agricultural byproducts.

This study establishes an optimized UAE protocol for polyphenol extraction from thinned peaches and identifies high-quality cultivar resources. By employing a Box–Behnken response surface methodology (RSM) with four critical parameters, ultrasonic time (X_1_), power (X_2_), liquid-to-material ratio (X_3_), and temperature (X_4_), we developed a predictive quadratic regression model to ascertain optimal extraction conditions. Through a comprehensive screening of 179 cultivars, we identified 20 elite cultivars that exhibited significantly enhanced levels of phenolics, flavonoids, and anthocyanins, as well as superior antioxidant capacities, as determined by FRAP, DPPH, and ABTS assays. This integrated approach, which combines process optimization, bioactivity assessment, and cultivars evaluation, provides both theoretical and technical foundations for the valorization of thinned peach resources in functional food and industrial applications.

## 2. Materials and Methods

### 2.1. Plant Materials

Freshly thinned peach fruit, encompassing 179 cultivars (including 16 wild accessions, 84 landraces, 76 cultivars, and 3 selections), were collected 4–5 weeks post-anthesis from the National Peach Germplasm Repository at the Jiangsu Academy of Agricultural Sciences (detailed germplasm information is available in Supplementary Table S1). Immediately following manual thinning, the fruit were snap-frozen in liquid nitrogen, cryo-ground into a fine powder using an IKA All Basic grinder (IKA Works GmbH, Staufen, Germany), and subsequently stored at −80 °C until further analysis.

### 2.2. Optimization of Conditions for Ultrasonic Assisted Extraction of Thinned Peach Polyphenolics (TPPs)

#### 2.2.1. Ultrasound-Assisted Extraction of Phenolic Compounds

One gram of thinned peach tissue was extracted using a pre-chilled methanol aqueous solution containing 0.1% HCl. The mixture was then subjected to sonication with an AS20500BDT ultrasonic instrument (Autoscience, Tianjin, China), with ultrasonic power, extraction temperature, and processing time varying according to experimental requirements, as detailed in the following section. Following sonication, the mixture was centrifuged at 8000× *g* for 10 min at 4 °C. The supernatant was collected and stored at −20 °C in a dark environment. It is essential to conduct the total phenol content analysis within 24 h.

#### 2.2.2. Single Factor Experiments

The experimental design evaluated five independent parameters using single-factor testing. The selected ranges for ultrasonic time (10, 15, 20, 25, 30, 35, 40, 45, and 50 min) and temperature (20, 30, 40, 50, 60, 70, and 80 °C) were informed by the work of Dai et al. (2021) [11]. Additionally, the parameters for methanol concentration (40, 50, 60, 70, 80, 90, and 100%, *v*/*v*), ultrasonic power (240, 300, 360, 420, 480, and 540 W), and liquid-to-solid ratio (10:1, 15:1, 20:1, 25:1, and 30:1 mL/g) were guided by Mihaylova et al. (2024) [17]. TPC was quantified spectrophotometrically using the Folin–Ciocalteu method as used by Dai et al. [11], with results expressed as grams of gallic acid equivalents per kilogram of fresh weight (g GAE/kg FW).

#### 2.2.3. Response Surface Design Experiments

Building upon the single-factor optimization studies, a four-factor, three-level Box–Behnken experimental design (BBD) was implemented using Design-Expert^®^ 9.0 software (Stat-Ease, Inc., Minneapolis, MN, USA) to systematically investigate variable interactions. The design matrix, encompassing ultrasonic power, temperature, treatment duration, and liquid-to-solid ratio, is detailed in Supplementary Table S2.

### 2.3. Quantitative Polyphenolic Analysis

#### 2.3.1. Total Phenolic Content (TPC)

The TPC was quantified using a modified Folin–Ciocalteu method, as adapted from Dai et al. [11]. A gallic acid standard solution at a concentration of 4 μg/mL was prepared, and serial aliquots ranging from 0.0 to 1.0 mL, in increments of 0.2 mL, were diluted to a total volume of 1.0 mL with distilled water in light-protected tubes. Each tube was then supplemented with 0.5 mL of Folin–Ciocalteu reagent and 3.0 mL of a 1.0 mol/L Na_2_CO_3_ solution, followed by vortex mixing. The mixtures were incubated for 120 min at 25 °C in the dark. Absorbance readings were taken at 760 nm using UV-Vis spectrophotometry (Shimadzu Corporation, Kyoto, Japan). The calibration curve obtained was linear, described by the equation A_760nm_ = 0.02237GA − 0.0393, with a coefficient of determination (R^2^) of 0.9912, where GA denotes the concentration of gallic acid in μg/mL. For sample analysis, 0.3 mL of polyphenolic extract from thinned fruit was treated with the same reagent addition and incubation protocols as the standard solutions. The TPC was quantified by interpolating the absorbance values of the samples against the established calibration curve. The results were reported as grams of gallic acid equivalents per kilogram of fresh weight (g GAE/kg FW).

#### 2.3.2. Total Flavonoid Content (TFC)

The TFC in thinned-fruit extracts was measured using a modified NaNO_2_-Al(NO_3_)_3_-NaOH colorimetric method. A rutin standard solution at a concentration of 0.4 mg/mL, prepared in a solvent mixture of HCl: methanol: water (1:70:29), was employed to construct a calibration curve. This curve was generated using aliquots ranging from 0 to 2 mL, which were adjusted to a total volume of 2 mL. The reaction protocol involved the sequential addition of 0.3 mL of 5% NaNO_2_, 0.3 mL of 10% Al(NO_3_)_3_, and 4 mL of 4% NaOH, followed by vortex mixing and a 20 min incubation period. The absorbance was measured at 510 nm using UV-Vis spectrophotometry. The resulting calibration curve demonstrated linearity, described by the equation A_510nm_ = 1.3325RT + 0.0065, R^2^ = 0.9987, where RT represents the rutin concentration. The same analytical procedure was applied to 0.6 mL of the thinned-fruit extract, and TFC values were determined by comparing the absorbance values of the samples against the established calibration curve. The results were expressed as grams of rutin equivalents per kilogram of fresh weight (g RTE/kg FW).

#### 2.3.3. Total Anthocyanin Content (TAC)

The TAC in thinned-fruit extracts was evaluated utilizing the H_2_SO_4_-C_8_H_8_O_3_ method as described by Zhang et al. [18]. A proanthocyanidin standard solution, with a concentration of 1 mg/mL, was prepared in methanol to establish the calibration curve. Aliquots ranging from 0 to 0.5 mL were adjusted to a total volume of 0.5 mL with methanol in light-protected tubes. Each tube was then supplemented with 1.5 mL of 30% H_2_SO_4_-CH_3_OH and 1.5 mL of 197.2 mmol/L C_8_H_8_O_3_, followed by thorough mixing and incubation at 30 °C for 20 min. Absorbance was measured at 500 nm using UV-Vis spectrophotometry. The calibration curve demonstrated a linear regression (A_500nm_ = 3.1648PA − 0.0039, R^2^ = 0.9995), where PA represents the concentration of proanthocyanidin. This method was similarly applied to 0.5 mL of thinned-fruit extract, and TAC values were determined by comparing the sample absorbance to the calibration curve. Results were expressed as grams of proanthocyanidin equivalents per kilogram of fresh weight (g PAE/kg FW).

### 2.4. Antioxidant Activity

#### 2.4.1. Ferric Reducing Antioxidant Power (FRAP)

The FRAP of thinned-fruit polyphenol extracts was evaluated using a modified method from Redondo et al. [19]. A calibration curve was created by diluting 0 to 50 μL of a 6 mmol/L Trolox solution to 50 μL with water in light-protected tubes. Then, 5 mL of FRAP reagent (0.1 mol/L acetate buffer, 10 mmol/L TPTZ, 20 mmol/L FeCl_3_ in a 10:1:1 ratio) was added. After incubating at 37 °C for 10 min and cooling, absorbance at 593 nm was measured. The calibration curve was linear: A_593nm_ = 7.4286(Trolox) − 0.0003 (R^2^ = 0.9995). Using the same procedure, 50 μL of thinned-fruit polyphenol extract was reacted with 5 mL FRAP reagent. FRAP values were obtained by interpolating sample absorbance against the calibration curve and expressed as mmol Trolox equivalents per kilogram of fresh weight (mmol TE/kg FW).

#### 2.4.2. 2,2′-Azino-di-3-ethylbenzthiazoline Sulfonic Acid (ABTS) Radical Scavenging Assay

The ABTS radical scavenging capacity of thinned-fruit polyphenol extracts was evaluated using a modified method from Re et al. [20]. A calibration curve was created by mixing serial aliquots of a 2 mmol/L Trolox solution with ABTS^+^ solution, prepared by combining 7 mmol/L ABTS and 2.5 mmol/L K_2_S_2_O_8_, incubated in the dark at 25 °C for 24 h, and diluted with ethanol to an absorbance of 1.0 at 734 nm. Absorbance was measured at 734 nm after a 5 min incubation. The calibration curve was linear: ΔA = 8.2762(Trolox) + 0.00117 (R^2^ = 0.9989), where (Trolox) is the concentration in mmol/L and ΔA = 1 − A_734nm_. For sample analysis, the same procedure was applied to thinned-fruit polyphenol extracts, and their ABTS radical scavenging capacity was determined by comparing sample absorbance to the calibration curve. Results were expressed as mmol Trolox equivalents per kilogram of fresh weight (mmol TE/kg FW).

#### 2.4.3. 2,2-Diphenyl-1-picrylhydrazyl (DPPH) Radical Scavenging Capacity

The DPPH radical scavenging activity of polyphenol extracts from thinned fruit was evaluated utilizing an advanced method adapted from Redondo et al. [19]. The analysis comprised two primary steps: the construction of a calibration curve and the quantification of the samples. For the calibration curve, serial dilutions of a 6 mmol/L Trolox standard were prepared, adjusted to a volume of 50 μL with water, and subsequently combined with 4 mL of a 0.3 mmol/L DPPH solution in ethanol. Following vortexing, the mixture was incubated for 30 min at 25 °C in the absence of light, and the absorbance was then recorded at 517 nm. The linear regression obtained was ΔA = 6.3491(Trolox) − 0.1039, with an R^2^ value of 0.9653. For the analysis of the samples, the same procedure was applied to the polyphenol extracts from thinned fruit, and their DPPH radical scavenging capacity was determined by comparing the sample absorbance to the calibration curve. The results were expressed as millimoles of Trolox equivalents per kilogram of fresh weight (mmol TE/kg FW).

### 2.5. Statistical Analysis

The experimental data were subjected to analysis utilizing Response Surface Methodology (RSM) via Design-Expert^®^ software (Version 13.0, State-Ease Inc., Minneapolis, MN, USA). Statistical significance was determined at a 95% confidence level (α = 0.05), with *p*-values less than 0.05 denoting significant model terms. A one-way analysis of variance (ANOVA) was performed to assess the goodness-of-fit between the experimental observations and the values predicted by the model, followed by verification of the normality and homoscedasticity of the residuals.

## 3. Results

### 3.1. Impact of Key Ultrasound Extraction Parameters on the Recovery Efficiency of Thinned Peach Polyphenols (TPPs)

**Figure 1:**
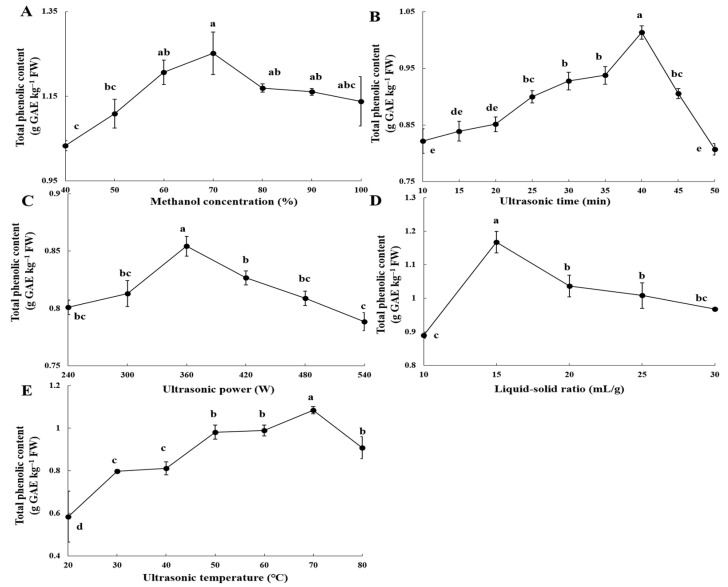
Effect of different ultrasound-assisted extraction parameters on the yield of total phenolic content from thinned peaches. Methanol concentration (**A**), ultrasonic time (**B**), ultrasonic power (**C**), liquid-to-solid ratio (**D**), and ultrasonic temperature (**E**). Data represent mean ± standard deviation (SD) (*n* = 3). Statistically significant differences are indicated by different letters (*p* < 0.05).

The UAE of TPC in this study demonstrated yield profiles that were contingent upon specific parameters, each exhibiting unique optimization thresholds (Figure 1). The extraction efficiency showed a concentration-dependent relationship with methanol, achieving a peak TPC yield of 1.14 g GAE/kg FW at a methanol concentration of 70%, after which the yields progressively declined (Figure 1A). The TPC yield exhibited a biphasic response to ultrasonic time, with a significant increase of 18.9% from 0 to 40 min, followed by a 20.4% decrease beyond 40 min, suggesting potential thermal degradation (Figure 1B). Optimal extraction efficiency was attained at an ultrasonic power of 360 W, resulting in a yield of 0.85 g GAE/kg FW, representing a 6.2% enhancement over the baseline conditions of 240 W. Applications exceeding 360 W led to a marked decline in yield, indicating the presence of energy saturation effects (Figure 1C). The liquid-to-solid ratio emerged as a pivotal factor in mass transfer processes, with optimal yields achieved at a ratio of 15:1 mL/g, resulting in a 23.9% enhancement (*p* < 0.05) compared to a 10:1 mL/g ratio. Ratios surpassing 15:1 mL/g led to an 11.2% decrease in yield, presumably due to solvent dilution effects (Figure 1D). Controlled heating significantly improved extraction efficiency up to 70 °C, with a 46.1% increase observed compared to 20 °C (*p* < 0.05); however, temperatures exceeding 70 °C caused a 16.2% decline in TPC (70–80 °C, *p* < 0.05), suggesting temperature-induced instability of phenolic compounds (Figure 1E). Analysis of variance (ANOVA) verified the statistically significant impact (*p* < 0.001) of ultrasonic time, power, liquid-solid ratio, and temperature on extraction efficiency, while methanol concentration also demonstrated a significant effect (*p* = 0.013) (Supplementary Table S3). Consequently, the identified inflection parameters for response surface modeling were as follows: ultrasonic time of 40 min, ultrasonic power of 360 W, a liquid-solid ratio of 15:1 mL/g, and an ultrasonic temperature of 70 °C.

### 3.2. Optimization of Extraction Conditions Using a Response Surface Model

The extraction process for TPPs was systematically optimized using RSM, specifically employing a Box–Behnken experimental design. As detailed in Table 1, the observed yield of TPC ranged from 0.764 to 1.196 g GAE/kg FW, indicating significant variability under the tested conditions. A second-order polynomial regression model was developed to quantify the interactions between parameters: Y = 1.08 + 0.0881X_1_ + 0.0284X_2_ + 0.0509X_3_ − 0.0283X_4_ − 0.0252X_1_X_2_ − 0.0531X_1_X_3_ + 0.0402X_1_X_4_ − 0.0018X_2_X_3_ − 0.0150X_2_X_4_ + 0.0026X_3_X_4_ − 0.0807X_1_^2^ − 0.0160X_2_^2^ − 0.0371 X_3_^2^ − 0.0367X_4_^2^.

The RSM analysis yielded a statistically validated model for predicting the extraction efficiency of TPC, with several critical findings. The quadratic model demonstrated high significance (*p* < 0.0001), affirming its appropriateness for optimization purposes. The non-significant lack of fit (*p* = 0.9797) further corroborated the model’s reliability. The coefficient of determination was R^2^ = 0.9459, indicating that 94.59% of the variability was explained by the model. The adjusted R^2^ was 0.8918, suggesting minimal overfitting and a robust predictive capability, as detailed in Table 2. The analysis also quantified the influence of parameters on TPPs yield through standardized coefficients: liquid-to-solid ratio (X_3_) and ultrasonic temperature (X_4_) were highly significant (*p* < 0.0001), followed by ultrasonic time (X_1_) (*p* = 0.005) and ultrasonic power (X_2_) (*p* = 0.0049).

**Figure 2:**
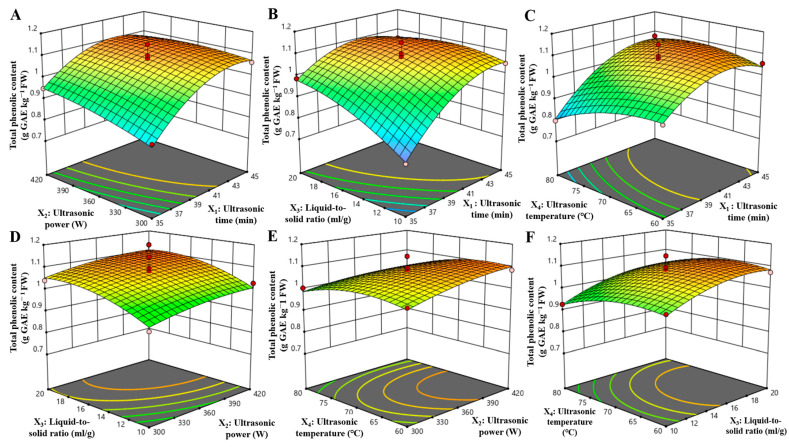
Response surface 3D plots showing the combined effects of ultrasound-assisted extraction parameters on the yield of total phenolic content from thinned peaches. (**A**) ultrasonic time and ultrasonic power; (**B**) ultrasonic time and liquid-to-solid ratio, (**C**) ultrasonic time and ultrasonic temperature, (**D**) ultrasonic power and liquid-to-solid ratio, (**E**) ultrasonic power and ultrasonic temperature, and (**F**) liquid-to-solid ratio and ultrasonic temperature. Color gradients: the colors represent the predicted TPC yield (mg GAE/g FW), with red/orange indicating higher yields and blue indicating lower yields. Circles: the scattered points denote experimental data points, while the surface plots reflect model-predicted values.

Response surface analysis utilizing three-dimensional surface plots (Figure 2E,F) identified ultrasonic temperature as the most influential factor affecting TPC. The highest polyphenol extraction was consistently achieved at the maximum temperature tested (70 °C) across ultrasonic power and liquid-to-solid ratios. Significant interactions among the process parameters were observed. First, a notable synergy between ultrasonic time and liquid-to-solid ratio was identified (*p* = 0.0029, Figure 2B). An increased liquid-to-solid ratio enhanced yield compensation at suboptimal ultrasonic durations (<41 min), although this compensatory effect diminished with longer extraction times (>41 min). Second, the interaction between ultrasonic time and temperature (*p* = 0.0163, Figure 2C) revealed risks of thermal degradation at shorter durations (<37 min), where elevated temperatures led to reduced yields. Conversely, extended processing times (>37 min) resulted in temperature-independent stabilization of polyphenol recovery. The model-derived optimized extraction parameters were determined to be a 45 min ultrasonic duration, 360 W ultrasonic power, a 15:1 mL/g liquid-to-solid ratio, and a 70 °C ultrasonic temperature. Experimental validation under these conditions yielded 1.12 ± 0.03 g GAE/kg FW (*n* = 3), achieving a 97.06% agreement with model predictions. This strong correlation between predicted and observed values confirms the robustness of the response surface methodology in process optimization.

Through response surface modeling, ultrasonic temperature was identified as the thermodynamically dominant factor at 70 °C. Multivariate interaction analysis further elucidated critical parameter interdependencies, including (1) liquid-solid ratio-mediated temporal compensation (*p* = 0.0029) and (2) duration-dependent thermal degradation thresholds (*p* = 0.0163). These findings culminated in the establishment of optimized operational thresholds (45 min, 360 W, 15:1 mL/g, 70 °C), achieving a near-perfect concordance between prediction and experimental outcomes (97.06%, 1.12 g GAE/kg FW), thereby validating the model’s precision in addressing extraction-process nonlinearities.

### 3.3. The Analysis of Polyphenolic Content in Thinned Peaches Across Various Germplasm Resources

**Figure 3:**
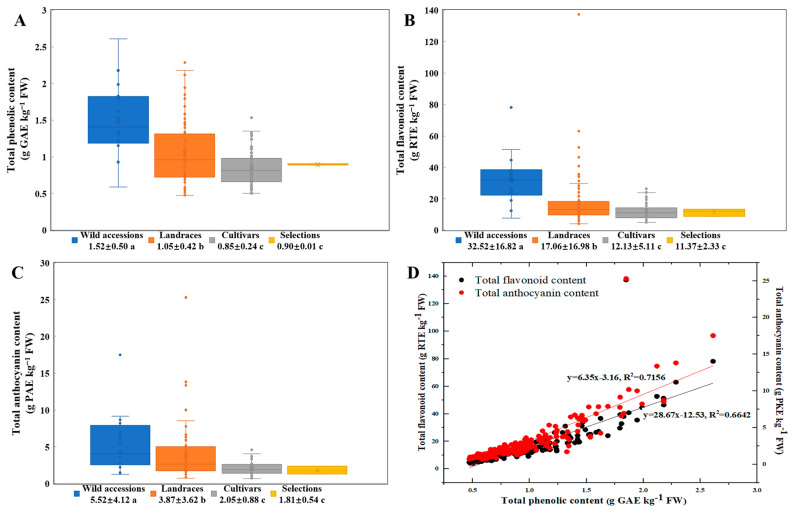
Compares the polyphenolic content among various types of thinned peach germplasm. (**A**) total phenolic content (TPC), (**B**) total flavonoid content (TFC), (**C**) total anthocyanin content (TAC), and (**D**) a bivariate correlation matrix with linear regression diagnostics for TPC, TFC, and TAC. Significant differences among different germplasm types are denoted by distinct lowercase letters (*p* < 0.05).

A comparative analysis of TPC, TFC, and TAC in TPPs across various germplasm resources reveals significant differences, as illustrated in Figure 3. Notably, TPC was significantly elevated in wild accessions (1.52 g GAE/kg FW) compared to other germplasm types. Specifically, it was approximately 1.4 times higher than in landraces (1.05 g GAE/kg FW) and 1.8 times higher than in both selections (0.90 g GAE/kg FW) and cultivars (0.85 g GAE/kg FW) (*p* < 0.05) (Figure 3A). Similarly, the TFC content in TPPs was highest in wild accessions (32.52 g RTE/kg FW), significantly surpassing that of landraces (17.06 g RTE/kg FW), cultivars (12.13 g RTE/kg FW), and selections (11.37 g RTE/kg FW) (*p* < 0.05). While no significant difference was observed between cultivars and selections, both were significantly lower than landraces (Figure 3B). Furthermore, the TAC in TPPs was markedly higher in wild accessions (5.52 g PAE/kg FW), being approximately 1.4 times higher than in landraces (3.87 g PAE/kg FW), 2.7 times higher than in cultivars (2.05 g PAE/kg FW), and 3.1 times higher than in selections (1.81 g PAE/kg FW), and no significant difference noted between cultivars and selections (Figure 3C).

Bivariate scatter plots with linear regression diagnostics demonstrated significant linear correlations between polyphenolic constituents in TPPs across germplasm resources: specifically, the relationship between TPC and TFC (y = 28.67x − 12.53, R^2^ = 0.6642), as well as between TPC and TAC (y = 6.35x − 3.16, R^2^ = 0.7156) (Figure 3D). These robust linear associations (R^2^ > 0.66) suggest that 66.4% to 71.6% of the variability in TFC and TAC can be statistically explained by TPC.

The phylogenetic conservation of phenolic biosynthesis was evident in wild peach germplasm, which demonstrated 1.4–3.1-fold higher TPC, TFC, and TAC compared to domesticated varieties (landraces, selections, and cultivars). Strong linear correlations between phenolic classes indicated a coordinated biosynthetic regulation of phenylpropanoid pathways during fruit thinning.

### 3.4. Comparative Analysis of Antioxidant Activity and Polyphenol-Antioxidant Correlations in TPPs Across Germplasm Types

**Figure 4:**
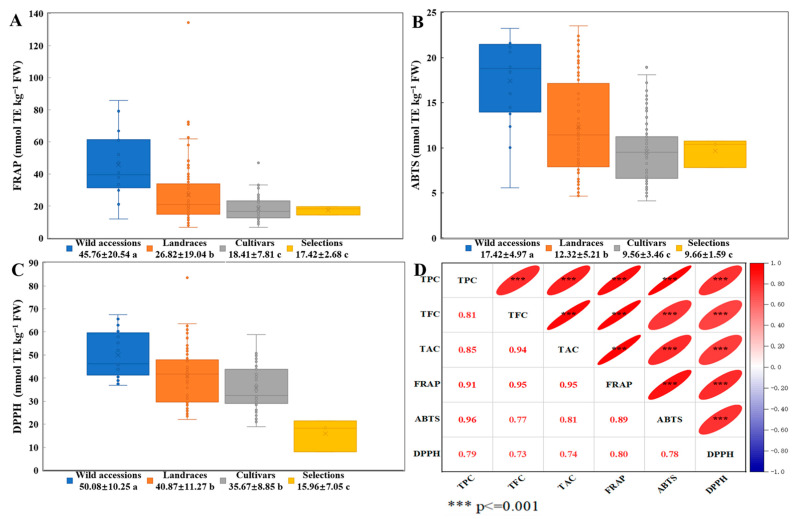
Comparative analysis of the antioxidant activities, exploring the relationships between polyphenolic content and antioxidant activities across different types of thinned peach germplasm. (**A**) Ferric reducing antioxidant power (FRAP), (**B**) ABTS radical scavenging activity, (**C**) DPPH radical scavenging activity, and (**D**) a multivariate correlation network analysis linking polyphenolic contents (TPC, TFC, and TAC) with antioxidant activities (FRAP, ABTS, and DPPH), red represents positive correlation, and blue represents negative correlation. The significance coding by letters is follows the conventions used in Figure 3 (*p* < 0.05).

A systematic evaluation was conducted to assess the antioxidant activity of TPPs derived from various germplasm types, alongside an exploration of the correlation between polyphenol content and antioxidant capacity, as illustrated in Figure 4. The FRAP assay indicated notable differences in total antioxidant capacity among the germplasm categories, with wild accessions exhibiting significantly higher activity (45.76 mmol TE/kg FW) relative to other germplasms. Specifically, wild accessions demonstrated 1.7-fold and 2.5-fold higher FRAP values than landraces (26.82 mmol TE/kg FW) and both cultivars (18.41 mmol TE/kg FW) and selections (17.42 mmol TE/kg FW), respectively. Landraces ranked second in FRAP values, surpassing cultivars and selections, which did not show significant differences from each other, thereby underscoring their intermediate phenotypic conservation (Figure 4A). In the ABTS radical scavenging assays, a similar pattern was observed, with wild accessions demonstrating the highest capacity (17.42 mmol TE/kg FW), significantly surpassing landraces (12.32 mmol TE/kg FW), cultivars (9.56 mmol TE/kg FW), and selections (9.66 mmol TE/kg FW). Notably, cultivars and selections exhibited comparable ABTS activity but remained statistically inferior to both wild accessions and landraces (Figure 4B). In parallel, the DPPH radical inhibition assays highlighted the superiority of wild accessions (50.08 mmol TE/kg FW), which outperformed landraces (40.87 mmol TE/kg FW), cultivars (35.67 mmol TE/kg FW), and selections (15.96 mmol TE/kg FW) (Figure 4C). These findings indicated that wild accessions possess the most robust antioxidant performance across all metrics, followed by landraces, whereas cultivars and selections exhibit comparatively lower activity. This reduced activity is likely attributable to the depletion of polyphenolic compounds during artificial breeding processes.

A correlation analysis of the polyphenol content and antioxidant activity in 179 peach thinned-fruit germplasm resources revealed highly significant positive correlations between TPC, TFC, and TAC with the antioxidant assays FRAP, ABTS, and DPPH (0.73 < *r* < 0.96, *p* < 0.001). Notably, the ABTS assay exhibited the strongest correlation with TPC (*r* = 0.96), while FRAP demonstrated high correlation coefficients with both TFC and TAC (*r* = 0.95) (Figure 4D). These results suggest that the polyphenol content in peach thinned fruit is a direct determinant of their antioxidant capacity, with wild accessions exhibiting enhanced antioxidant activity due to their higher polyphenol retention.

The wild thinned peach germplasm demonstrated a phylogenetically stratified superiority in antioxidant properties, surpassing landraces by 1.7 to 2.5 times and cultivars/selections by 2.5 to 3.1 times. This was evidenced by dose-dependent linear correlations (*r* = 0.73–0.96, *p* < 0.001) between polyphenolic levels (TPC/TFC/TAC) and antioxidant metrics (FRAP/ABTS/DPPH).

### 3.5. High-Quality Germplasm Resources of Peach Thinned Fruits

This study conducted a comprehensive evaluation of polyphenol content and antioxidant activity in TPPs to identify 20 superior peach germplasm resources, as detailed in Table 3. These resources encompass eight wild accessions, eleven landraces, and one cultivar. Among these, the landrace ‘Early White Blossom Peach’ exhibited exceptional characteristics, including the highest TFC of 137.31 g RTE/kg FW, TAC of 25.30 g PAE/kg FW, and superior antioxidant performance as indicated by a FRAP of 134.46 mmol TE/kg FW, an ABTS radical scavenging capacity of 23.50 mmol TE/kg FW, and a DPPH radical scavenging capacity of 83.58 mmol TE/kg FW. Furthermore, the wild accession ‘Gansu Peach 2’ demonstrated the highest TPC of 2.61 g GAE/kg FW in its TPPs and a notably high ABTS radical scavenging capacity of 23.24 mmol TE/kg FW.

The screening of germplasm identified 20 elite peach resources (8 wild, 11 landraces, 1 cultivar) exhibiting superior phenolic-antioxidant synergy. Notable examples include ‘Early White Blossom Peach’ and the wild ‘Gansu Peach 2’, which illustrate the advantages of landrace-wild hybrid in maintaining nutraceutical potency despite the genetic bottlenecks associated with domestication.

## 4. Discussion

Peach thinning fruit have emerged as a significant source of bioactive polyphenols, with contemporary research concentrating on three primary dimensions: (1) detailed phytochemical profiling [21], (2) quantitative evaluation of antioxidant capacity [10], and (3) mechanistic elucidation of biological activities [6,22]. Despite notable advancements in these domains, the optimization of extraction methodologies remains a crucial area for further investigation. The efficiency of phenolic compound extraction is predominantly influenced by solvent polarity. Comparative analyses have demonstrated that methanol outperforms water and ethanol, facilitating both enhanced polyphenol recovery and superior preservation of antioxidant activity [16]. Conventional extraction using 80% methanol for 35 min yields a TPC of 0.83 g GAE/kg FW [23]. However, our optimization efforts have identified an optimal TPC of 1.14 g GAE/kg FW at a 70% methanol concentration (Figure 1A), consistent with the “like dissolves like” principle applicable to low molecular weight polar phenolics. Furthermore, temperature is a critical factor influencing the efficiency of polyphenol extraction. Our results indicate that increasing the temperature from 20 °C to 70 °C resulted in a 46.09% increase in TPC, from 0.58 to 1.08 g GAE/kg FW. This finding aligns with previous studies, which reported a 113% improvement in TFC, from 38 to 81 mg QE/100 g, when the temperature was increased from 25 °C to 70 °C [16]. However, thermal degradation was observed at temperatures exceeding 80 °C (Figure 1E). The enhancement of extraction efficiency with increasing temperature can be attributed to the softening of plant tissue, which facilitates the release of polyphenols by weakening phenol-protein and phenol-polysaccharide interactions [24], as well as the increased solubility of glycosides, which improves the extraction of flavonoid glycosides at higher temperatures [25]. These mechanisms collectively elucidate the observed positive correlation between temperature and phenolic yield within the optimal range of 20 °C to 70 °C.

The UAE has emerged as a leader in the development of highly efficient and mild processing technologies for polyphenol extraction, presenting significant advantages over traditional solvent-based methods. Notable benefits include increased polyphenol yields, ranging from 34.9% to 135.5%, preservation of the integrity of bioactive compounds [26,27], and selective enrichment of functional components, such as chlorogenic acid, in comparison to microwave-assisted extraction [28]. Furthermore, a comparative techno-functional analysis demonstrated the superior efficacy of UAE in polyphenol recovery from peach pomace, achieving maximal yields (0.45 g GAE/kg FW) and antioxidant retention (51.9%) in contrast to high-pressure (500 MPa/10 min: 0.40 g GAE/kg FW) and microwave-assisted (90 s: 0.34 g GAE/kg FW) extraction methods [29], Consequently, UAE is established as the preferred method for further optimization due to its operational simplicity and extraction efficiency. Systematic investigations have identified critical parameters for UAE, including ultrasonic duration, temperature, power, and solid-to-liquid ratio, which collectively enhance extraction efficiency [15]. Under optimized conditions, 25 min, 50 °C, 147 W, and a 12:1 mL/g solid-to-liquid ratio, Dai et al. [11] reported a polyphenol yield of 1.59 g GAE/kg FW in thinned peaches. Recent studies by Mihaylova et al. [17] on the Bulgarian peach cultivar ‘Filina’ demonstrated a TPC of 1.06 g GAE/kg FW under extended UAE conditions (39.33 min, 70 °C, 20:1 mL/g). Our optimized protocol, employing 45 min, 70 °C, 360 W, and a 15:1 mL/g ratio, achieved a TPC of 1.12 g GAE/kg FW, thereby confirming the consistency of technical parameters across various studies. These variations in optimal conditions and yields can largely be attributed to differences in fruit material properties (e.g., cultivar and polyphenol composition). The consistency in UAE performance across studies highlights its suitability for scaling up, offering adjustable parameters for tailored extraction, compatibility with industrial equipment, and sustainable valorization of peach thinning byproducts into high-value nutraceutical and pharmaceutical ingredients. By enhancing mass transfer through mechanical vibration and cavitation effects, UAE presents a robust and efficient method for transforming underutilized agricultural waste into bioactive-rich extracts.

Polyphenols, a category of plant secondary metabolites distinguished by hydroxylated aromatic rings, demonstrate dynamic accumulation patterns throughout the development of peach fruit [19]. Quantitative analyses indicate a substantial 95% reduction in TPC and TFC from the thinning stage (48 days post-full bloom) to commercial harvest, with concentrations decreasing from 1.54 and 1.19 g GAE/kg FW (in peach and nectarine flesh) to 0.11 and 0.092 g GAE/kg FW, respectively [30]. Metabolomic profiling has identified NCGA, catechin, and CGA as the predominant monomeric phenolics, collectively accounting for 88.80–95.45% of polyphenols in thinned fruit and 70.18–84.56% in mature fruit [21]. Notably, their concentrations at the thinning stage are 6.4–20.7 times higher for NCGA, 3.3–14.2 times higher for catechin, and 4.1–11.4 times higher for CGA compared to levels at maturity [6,10]. This decline is strongly correlated with polyphenol oxidase (PPO)-mediated oxidation and polymerization processes [30]. Additionally, significant varietal differences are evident, with the mid-season variety ‘Gergana’ exhibiting a TPC of 0.79 g/kg FW, which is 8.78 times higher than that of the early-season variety ‘Laskava’ (0.09 g/kg FW) [31]. Cultivars with red flesh demonstrate a greater abundance of phenolics compared to their white or yellow-fleshed counterparts [32]. Moreover, among 20 evaluated cultivars, the wild-derived ‘WB 258’ exhibits the highest TPC at 31.16 g/kg DM, which is 4.3 times greater than that of ‘Madison’ (7.23 g/kg DM) [14]. Wild accessions consistently outperform landraces and cultivars, as illustrated in Figure 3. The landrace ‘Early White Blossom Peach’ displays exceptional levels of TFC (137.72 g RTE/kg FW) and anthocyanins (25.30 g PAE/kg FW), which are 34.24 and 37.00 times higher than those in ‘Shiwo Honey Peach’, respectively (Table 3). Moreover, the observed strong correlations between TPC and TFC (R^2^ = 0.66), as well as between TPC and TAC (R^2^ = 0.72), indicate a coordinated biosynthetic pathway. Germplasms with high polyphenol content exhibit increased resistance to *Monilinia* spp., with wild accessions demonstrating a 60–75% reduction in brown rot incidence compared to cultivated varieties [33]. This resistance is attributed to two primary mechanisms: (1) direct antifungal activity through CGA and NCGA-mediated inhibition of fungal melanin biosynthesis [34], and (2) modulation of oxidative stress responses [35]. These findings underscore the potential of thinning-stage fruits and high-phenolic genotypes, such as the wild accession ‘Gansu Peach 2’ and the landrace ‘Early White Blossom Peach’, as valuable sources of polyphenols. Their strategic utilization could facilitate the development of nutraceuticals and biocontrol agents, while simultaneously addressing waste challenges within the peach industry.

The antioxidant capacity of peach polyphenols is attributed to their unique molecular structure, particularly the synergistic electronic interactions between aromatic rings and multiple phenolic hydroxyl groups [19]. This structural arrangement imparts significant radical-scavenging activity, facilitating photoprotective and oxidative stress-modulating effects. Phenolic acids, especially NCGA, catechin, and CGA, are the primary contributors to the antioxidant activity in peach fruit, surpassing the efficacy of vitamin C and carotenoids [9,21,36,37]. Peaches and nectarines at the thinning stage exhibit 1.3–11.2 times greater antioxidant capacity, as measured by FRAP, DPPH, and ABTS assays, compared to mature fruit [10]. Moreover, red-fleshed cultivars demonstrate superior antioxidant performance, with FRAP values 5.8- and 6.2-fold higher and relative antioxidant capacity (RAC) 3.8- and 4.6-fold higher than those of white- and yellow-fleshed cultivars, respectively [38]. In this study, a comprehensive analysis of 179 peach thinning-fruit resources demonstrated that wild germplasms exhibit significantly greater antioxidant activity compared to landraces, cultivars, and selections (Figure 3). While utilizing three electron transfer (ET)-based assays, FRAP, DPPH, and ABTS, we observed significant inter-correlations (0.78 < *r* < 0.89, *p* < 0.001) and consistent trends across all cultivars (Figure 4D), suggesting genetic determinants of antioxidant capacity. The established correlation between ET-based methods, FRAP, DPPH, and ABTS, and oxygen radical absorbance capacity (ORAC) values in peach polyphenols [8,39], further validates the reliability of our screening results and demonstrates that ET-based detection systems can effectively reflect the biological antioxidant potential of peach polyphenols. Notably, the landrace ‘Early White Blossom Peach’ displayed exceptional antioxidant properties, with FRAP, ABTS, and DPPH radical scavenging activities measuring 134.46 mmol TE/kg FW, 23.50 mmol TE/kg FW, and 83.58 mmol TE/kg FW, respectively (Table 3). Moreover, correlation analyses confirmed strong positive relationships (*p* < 0.001) between TPC, TFC, and TAC in thinning fruit and all antioxidant indices (FRAP/ABTS/DPPH). The significant antioxidant capacity of polyphenols derived from peach thinning fruit highlights their diverse applications, including their potential use as nutraceuticals for cancer prevention [40], anti-inflammatory treatments [12,13], and obesity management strategies [6,14,41], as well as their incorporation into high-end skincare products [22]. These results highlight the potential of thinning-stage fruit and high-antioxidant germplasms, such as red-fleshed cultivars and wild accessions, for utilization in value-added applications within the nutraceutical, cosmeceutical, and functional food industries, while also addressing issues related to agricultural waste.

## 5. Conclusions

This study developed an optimized ultrasound-assisted extraction method for recovering polyphenols from peach thinning byproducts. A systematic evaluation of 179 germplasm resources identified wild accessions as superior sources of bioactive compounds, exhibiting enhanced antioxidant properties compared to domesticated varieties. The demonstrated linkage between phytochemical diversity and bioactivity underscores their potential for valorization in functional food applications. Future research should focus on elucidating the molecular mechanisms underlying antioxidant biosynthesis in wild accessions through integrated genomics-metabolomics approaches and engineering sustainable extraction platforms for the industrial-scale production of thinned peach-derived nutraceuticals.

## Figures and Tables

**Table 1:** Design and results of Box–Behnken response surface experiment.

Number	X_1_/Ultrasonic Time (min)	X_2_/Ultrasonic Power (W)	X_3_/Liquid-to-Solid (mL/g)	X_4_/Ultrasonic Temperature (°C)	Total Phenolic Content (g GAE kg^−1^ FW)
1	40 (0)	360 (0)	10:1 (−1)	60 (−1)	0.994 ± 0.003 ^hij^
2	40 (0)	420 (1)	15:1 (0)	80 (1)	1.006 ± 0.001 ^ghi^
3	35 (−1)	360 (0)	15:1 (0)	60 (−1)	0.938 ± 0.001 ^kl^
4	35 (−1)	360 (0)	10:1 (−1)	70 (0)	0.764 ± 0.000 ^n^
5	40 (0)	360 (0)	20:1 (1)	80 (1)	1.015 ± 0.002 ^fghi^
6	40 (0)	420 (1)	10:1 (−1)	70 (0)	1.027 ± 0.001 ^defghi^
7	45 (1)	420 (1)	15:1 (0)	70 (0)	1.052 ± 0.001 ^cdefgh^
8	35 (−1)	360 (0)	20:1 (1)	70 (0)	0.986 ± 0.003 ^ijk^
9	35 (−1)	420 (1)	15:1 (0)	70 (0)	0.949 ± 0.001 ^jkl^
10	40 (0)	420 (1)	20:1 (1)	70 (0)	1.131 ± 0.000 ^ab^
11	45 (1)	360 (0)	10:1 (−1)	70 (0)	1.047 ± 0.000 ^cdefgh^
12	40 (0)	300 (−1)	15:1 (0)	80 (1)	1.003 ± 0.003 ^ghij^
13	45 (1)	300 (−1)	15:1 (0)	70 (0)	1.060 ± 0.002 ^cdefg^
14	35 (−1)	300 (−1)	15:1 (0)	70 (0)	0.856 ± 0.000 ^m^
15	45 (1)	360 (0)	15:1 (0)	60 (−1)	1.056 ± 0.002 ^cdefg^
16	40 (0)	360 (0)	15:1 (0)	70 (0)	1.082 ± 0.001 ^bcd^
17	45 (1)	360 (0)	15:1 (0)	80 (1)	1.077 ± 0.000 ^bcde^
18	40 (0)	360 (0)	15:1 (0)	70 (0)	1.045 ± 0.002 ^cdefghi^
19	40 (0)	360 (0)	10:1 (−1)	80 (1)	0.927 ± 0.001 ^l^
20	40 (0)	300 (−1)	10:1 (−1)	70 (0)	0.929 ± 0.001 ^l^
21	40 (0)	360 (0)	15:1 (0)	70 (0)	1.034 ± 0.002 ^defghi^
22	40 (0)	300 (−1)	20:1 (1)	70 (0)	1.040 ± 0.000 ^cdefghi^
23	40 (0)	360 (0)	15:1 (0)	70 (0)	1.196 ± 0.002 ^a^
24	40 (0)	300 (−1)	15:1 (0)	60 (−1)	1.021 ± 0.002 ^efghi^
25	40 (0)	420 (1)	15:1 (0)	60 (−1)	1.084 ± 0.001 ^bcd^
26	40 (0)	360 (0)	20:1 (1)	60 (−1)	1.072 ± 0.003 ^cdef^
27	40 (0)	360 (0)	15:1 (0)	70 (0)	1.095 ± 0.037 ^abc^
28	45 (1)	360 (0)	20:1 (1)	70 (0)	1.056 ± 0.001 ^cdefg^
29	35 (−1)	360 (0)	15:1 (0)	80 (1)	0.798 ± 0.001 ^n^

Note: the result was performed using Design Expert 13.0 software (State-Ease Inc., Minneapolis, MN, USA). Data represents mean values ± standard deviation (*n* = 3). Mean with different superscript letters within a column differ significantly (*p* < 0.05).

**Table 2:** Results of ANOVA from response surface experiment.

Source	Sum of Squares	df	Mean Square	F-Value	*p*-Value	Significant
Model	0.2126	14	0.0152	17.49	<0.0001	**
X_1_ (Ultrasonic time)	0.0932	1	0.0932	107.36	0.005	**
X_2_ (Ultrasonic power)	0.0097	1	0.0097	11.12	0.0049	**
X_3_ (Liquid-to-solid ratio)	0.0311	1	0.0311	35.83	<0.0001	**
X_4_ (Ultrasonic temperature)	0.0096	1	0.0096	11.04	<0.0001	**
X_1_X_2_	0.0025	1	0.0025	2.92	0.1098	
X_1_X_3_	0.0113	1	0.0113	13.00	0.0029	**
X_1_X_4_	0.0065	1	0.0065	7.45	0.0163	*
X_2_X_3_	0.0000	1	0.0000	0.0147	0.9052	
X_2_X_4_	0.0009	1	0.0009	1.03	0.3263	
X_3_X_4_	0.0000	1	0.0000	0.0320	0.8606	
X_1_^2^	0.0422	1	0.0422	48.62	<0.0001	**
X_2_^2^	0.0017	1	0.0017	1.92	0.1873	
X_3_^2^	0.0089	1	0.0089	10.31	0.0063	**
X_4_^2^	0.0087	1	0.0087	10.04	0.0068	**
Residual	0.0122	14	0.0009			
Lack of Fit	0.0042	10	0.0004	0.2088	0.9797	
Cor Total	0.2248	28				
R^2^ = 0.9459, R^2^_adj_ = 0.8918, R^2^_pre_ = 0.8376						

Note: * indicates (*p* < 0.05), ** (*p* < 0.01).

**Table 3:** Comparative analysis of phytochemical contents and antioxidant activities in top 20 polyphenol-rich thinned peach cultivars: total phenolics, flavonoids, anthocyanins, ABTS/DPPH radical scavenging, and FRAP reducing power.

Germplasm Type	Name	TPC	TFC	TAC	FRAP	ABTS	DPPH
		g GAE kg^−1^ FW	g RTE kg^−1^ FW	g PAE kg^−1^ FW	mmol TE kg^−1^ FW	mmol TE kg^−1^ FW	mmol TE kg^−1^ FW
WA	Gansu Peach 2	2.6 ± 0.1 ^a^	78.2 ± 10.4 ^b^	17.5 ± 1.6 ^b^	85.8 ± 11.0 ^b^	23.2 ± 0.1 ^a^	67.5 ± 0.1 ^b^
LD	Nanshan Sweet Peach 1	2.3 ± 0.1 ^b^	63.0 ± 6.7 ^c^	13.8 ± 0.8 ^c^	72.4 ± 2.2 ^cd^	22.6 ± 0.2 ^b^	63.4 ± 1.1 ^c^
LD	Yanwohong	2.2 ± 0.1 ^bc^	46.4 ± 2.2 ^de^	8.5 ± 0.5 ^ef^	58.1 ± 3.9 ^fgh^	22.1 ± 0.2 ^bc^	61.0 ± 0.9 ^de^
WA	Fujian Hairy Peach 1	2.2 ± 0.1 ^bc^	51.3 ± 7.5 ^d^	8.7 ± 0.7 ^ef^	79.2 ± 8.4 ^bc^	21.6 ± 0.2 ^cd^	62.9 ± 1.3 ^cd^
LD	Nanshan Sweet Peach	2.1 ± 0.1 ^cd^	52.7 ± 2.2 ^d^	13.4 ± 0.3 ^c^	70.9 ± 10.2 ^cde^	22.4 ± 0.1 ^b^	62.6 ± 0.6 ^cd^
WA	Guanghetao	2.0 ± 0.1 ^de^	44.5 ± 2.5 ^def^	8.2 ± 0.2 ^ef^	66.8 ± 3.7 ^def^	21.7 ± 0.3 ^cd^	65.6 ± 0.8 ^b^
LD	Purple Nectarine 9	2.0 ± 0.1 ^ef^	35.5 ± 3.6 ^fghi^	10.0 ± 0.1 ^d^	62.0 ± 3.0 ^ef^	21.4 ± 0.3 ^cd^	59.5 ± 0.2 ^e^
LD	Lianyungang Winter Peach	1.9 ± 0.2 ^ef^	40.8 ± 10.5 ^efg^	10.2 ± 1.7 ^d^	62.8 ± 4.8 ^def^	21.9 ± 0.4 ^bc^	57.3 ± 0.8 ^f^
LD	Early White Blossom Peach	1.9 ± 0.1 ^efg^	137.3 ± 12.5 ^a^	25.3 ± 0.0 ^a^	134.5 ± 2.5 ^a^	23.5 ± 0.1 ^a^	83.6 ± 0.6 ^a^
WA	Red Flower Mountain Peach	1.8 ± 0.1 ^efg^	38.2 ± 3.9 ^efgh^	6.9 ± 0.5 ^ghi^	61.1 ± 7.0 ^efg^	21.1 ± 0.9 ^de^	52.0 ± 2.7 ^h^
WA	Thai Hairy Peach	1.8 ± 0.1 ^fg^	32.9 ± 0.7 ^ghij^	6.6 ± 0.2 ^hi^	47.1 ± 2.7 ^ij^	20.65 ± 0.39 ^ef^	46.1 ± 1.4 ^j^
WA	Guanghetao 24-1	1.8 ± 0.1 ^fg^	39.5 ± 1.6 ^efgh^	9.2 ± 0.6 ^de^	61.5 ± 2.6 ^efg^	21.60 ± 0.18 ^cd^	60.3 ± 1.1 ^e^
LD	Zhanghuang 3	1.8 ± 0.2 ^fg^	29.6 ± 4.0 ^hij^	7.8 ± 0.8 ^fgh^	45.5 ± 6.9 ^ij^	20.70 ± 0.80 ^ef^	54.9 ± 0.5 ^g^
LD	Yexian Yellow Peach 8	1.7 ± 0.1 ^gh^	24.1 ± 2.1 ^j^	7.9 ± 0.3 ^fg^	49.2 ± 6.7 ^hi^	20.13 ± 0.28 ^fg^	54.1 ± 0.4 ^g^
WA	Hairy Peach	1.6 ± 0.1 ^hi^	36.7 ± 2.8 ^efgh^	4.2 ± 0.5 ^j^	37.9 ± 2.5 ^j^	18.92 ± 0.76 ^hi^	40.5 ± 0.4 ^k^
LD	Purple Nectarine 5	1.6 ± 0.1 ^hi^	27.2 ± 2.4 ^ij^	7.8 ± 0.4 ^fg^	48.7 ± 2.9 ^hi^	19.91 ± 0.27 ^g^	50.6 ± 1.9 ^hi^
LD	Red Leaf Winter Peach	1.6 ± 0.1 ^hi^	26.6 ± 1.6 ^ij^	6.9 ± 0.3 ^ghi^	48.5 ± 4.2 ^hi^	19.67 ± 0.17 ^g^	48.6 ± 2.5 ^i^
CV	Shuangbai	1.5 ± 0.1 ^hi^	24.1 ± 1.6 ^j^	3.7 ± 0.3 ^j^	46.9 ± 2.0 ^ij^	17.21 ± 0.26 ^j^	58.8 ± 1.9 ^ef^
LD	Rugao Purple Nectarine 3	1.5 ± 0.1 ^i^	24.9 ± 0.4 ^j^	7.8 ± 0.1 ^fgh^	44.3 ± 3.8 ^ij^	18.03 ± 0.13 ^i^	50.7 ± 0.1 ^hi^
WA	Red Leaf Flat Peach peach	1.5 ± 0.1 ^i^	35.2 ± 4.5 ^fghi^	6.2 ± 0.5 ^i^	52.1 ± 0.6 ^ghi^	20.62 ± 0.02 ^ef^	45.8 ± 0.8 ^j^

Note: Each value is expressed as the mean of three values ± standard deviation (*n* = 3), and in each cell, different superscript letters within the same cell indicate significant differences (*p* < 0.05). Abbreviations: TPC, total phenolic content; TFC, total flavonoid content; TAC, total anthocyanin content; FRAP, ferric reducing antioxidant power; ABTS, 2,2′-azino-bis (3-ethylbenzothiazoline-6-sulfonic acid); DPPH, 2,2-diphenyl-1-picrylhydrazyl; WA, wild accession; LD, landraces; CV, cultivars; ST, selections.

## Data Availability

The original contributions presented in the study are included in the article/Supplementary Materials, further inquiries can be directed to the corresponding author.

## Supplementary Materials

The following supporting information can be downloaded at: https://www.mdpi.com/article/10.3390/foods14111897/s1, Table S1: Code, Name, Germplasm type, Fruit type and Country of the germplasms used; Table S2: Code and decoded levels of independent variables used in the RSM design; Table S3: One-way ANOVA analysis.
