# Supplementary material for: Ultrasound-Assisted Extraction Optimization and Cultivar Screening for Polyphenol Recovery from Thinned Peach Fruit: A Comprehensive Evaluation of 179 Cultivars
Source: Foods. 2025 May 27;14(11):1897. doi: 10.3390/foods14111897 (PMC12155168; doi:10.3390/foods14111897)
Supplement: Supplementary file 1 [file foods-14-01897-s001.zip › foods-3632626-supplementary.pdf]

Supplementary Table S1. Code, Name, Germplasm type, Fruit type and Country of the germplasms used.

**Code**	**Name**	**Germplasm type**	**Fruit type**	**Country**
1	Jianli Hairy Peach	Wild accessions	Peach	China
2	Early White Blossom Peach	Landraces	Peach	China
3	Pingchang Hairy Peach	Wild accessions	Peach	China
4	Red Leaf Flat Peach	Wild accessions	Flat peach	China
5	Yunlong Peach	Cultivars	Nectarine	China
6	Lijiang Peach	Landraces	Peach	China
7	Fragrant Peach	Cultivars	Peach	China
8	Late White Blossom Peach	Landraces	Peach	China
9	Late White Peach	Cultivars	Peach	China
10	21st Century Peach	Cultivars	Peach	China
11	Xipu Giant Jade Dew Peach	Cultivars	Peach	China
12	Yingqing Peach	Cultivars	Peach	China
13	Late Round Honey Peach	Cultivars	Peach	China
14	Longhua Honey Peach	Landraces	Peach	China
15	Hanlu Honey Peach	Landraces	Peach	China
16	Jingchun Peach	Cultivars	Peach	China
17	Giant Jade Dew Co60 Peach	Cultivars	Peach	China
18	Early Freestone Jade Dew Peach	Landraces	Peach	China
19	Jiqing Red Peach	Landraces	Peach	China
20	Lianyungang Winter Peach	Landraces	Peach	China
21	Jinjiuhong Peach	Cultivars	Peach	China
22	Red-Flesh Peach 1	Landraces	Peach	China
23	Touxin Red	Landraces	Peach	China
24	GYT026 Peach	Landraces	Peach	China
25	Chenpu Flat Peach	Landraces	Flat peach	China
26	124 Flat Peach	Cultivars	Flat peach	China
27	Nong Shen	Cultivars	Flat peach	America
28	Early Yellow Flat Peach	Cultivars	Flat peach	China
29	Early Dew Flat Peach	Cultivars	Flat peach	China
30	Chunyan	Cultivars	Peach	China
31	Xiahui 2	Cultivars	Peach	China
32	Yuandong White Peach	Cultivars	Peach	China
33	Red Honeydew Peach	Cultivars	Peach	China

34	Wrinkle-Leaf Yellow Dew Peach	Cultivars	Peach	China
35	Hujing Honey Peach	Cultivars	Peach	China
36	Datuan Honey Peach	Landraces	Peach	China
37	Snow White Peach	Landraces	Peach	China
38	Huiyu Dew	Cultivars	Peach	China
39	Yuhua 3	Cultivars	Peach	China
40	Fenglu	Cultivars	Peach	China
41	Dianhua White Phoenix Peach	Landraces	Peach	China
42	Yangshan Peach	Landraces	Peach	China
43	Shangshan Giant Jade Dew Peach	Cultivars	Peach	China
44	Fenghua Jade Dew (Late)	Landraces	Peach	China
45	Jingtang Late Honey Peach	Cultivars	Peach	China
46	Xiwei Giant Yellow Peach	Cultivars	Peach	Japan
47	Changyin 2	Cultivars	Peach	Japan
48	99-13-9 Peach	Selections	Peach	China
49	Yuhua Dew	Cultivars	Peach	China
50	Yuanchun White	Landraces	Peach	China
51	Yangtao 97	Cultivars	Peach	China
52	Renpu Honey Peach	Landraces	Peach	China
53	Late Giant Honey Peach	Cultivars	Peach	China
54	Tangxing White Blossom Peach	Landraces	Peach	China
55	Late Jade Dew Peach	Landraces	Peach	China
56	Changzhou White Blossom Peach	Landraces	Peach	China
57	Qiyuan Honey Peach	Landraces	Peach	China
58	62-43 Peach	Selections	Peach	China
59	Xiahui 8	Cultivars	Peach	China
60	Early White Phoenix 79	Cultivars	Peach	China
61	Small Red Blossom Peach	Landraces	Peach	China
62	Big Red Blossom Peach	Landraces	Peach	China
63	Changyin 1	Cultivars	Peach	Japan
64	Giant Treasure Red Moon Peach	Cultivars	Peach	Japan
65	Japanese White Peach	Cultivars	Nectarine	Japan
66	Masahime	Cultivars	Peach	Japan
67	Yuzora	Cultivars	Peach	Japan
68	Famous White Peach	Cultivars	Peach	South Korea

69	Meixiang	Cultivars	Peach	Japan
70	Fengbai	Cultivars	Peach	China
71	Phoenix Honey Peach	Landraces	Peach	China
72	Xiahui 6	Cultivars	Peach	China
73	Beijing Hairy Peach	Wild accessions	Peach	China
74	Xiahui 8	Cultivars	Peach	China
75	Qin King Peach	Cultivars	Peach	China
76	Zhonghua Longevity Peach	Cultivars	Peach	China
77	Lianghe Honey Peach	Landraces	Peach	China
78	Late Honey Peach	Cultivars	Peach	China
79	Chongyang Red	Cultivars	Peach	China
80	Ganxuan 2	Cultivars	Peach	China
81	Liangji	Cultivars	Peach	Japan
82	Ganxuan 4	Landraces	Peach	China
83	Ganxuan 1	Landraces	Peach	China
84	Baili	Cultivars	Peach	Japan
85	Yanhong	Cultivars	Peach	China
86	Huayu	Cultivars	Peach	China
87	Wanhongmi	Cultivars	Peach	China
88	Shuangbai	Cultivars	Peach	China
89	Zitao	Landraces	Peach	China
90	Hongburuan	Cultivars	Peach	America
91	Nakatsukasa White Peach	Cultivars	Peach	Japan
92	Xiahui 7	Selections	Peach	China
93	Lingfeng	Cultivars	Peach	Japan
94	Xianyinu	Cultivars	Peach	Japan
95	Chiyu	Cultivars	Peach	Japan
96	Ba 9	Cultivars	Peach	Brazil
97	Nonglin 90	Cultivars	Peach	Japan
98	Nonglin 89	Cultivars	Peach	Japan
99	Dayu Baifeng	Cultivars	Peach	Japan
100	Zhaohui	Landraces	Peach	China
101	Ganxuan 3	Landraces	Peach	China
102	Xiahui 5	Cultivars	Peach	China
103	Guanghetao	Wild accessions	Peach	China
104	Guanghetao 24-1	Wild accessions	Peach	China

105	Shaanxi Peach Badan	Wild accessions	Peach	China
106	Peach Badan	Wild accessions	Peach	China
107	Fujian Hairy Peach 1	Wild accessions	Peach	China
108	Tsukuba 5	Cultivars	Peach	Japan
109	Luoge Red Leaf Double	Wild accessions	Peach	America
110	Red Flower Mountain Peach	Wild accessions	Peach	China
111	Thai Hairy Peach	Wild accessions	Peach	Thailand
112	Gansu Peach 2	Wild accessions	Peach	China
113	Hairy Peach	Wild accessions	Peach	China
114	Gansu Peach	Wild accessions	Peach	China
115	Gaobeibi Hairy Peach 1	Wild accessions	Peach	China
116	Jinxia Flat Peach	Cultivars	Peach	China
117	Yuxia Flat Peach	Cultivars	Flat peach	China
118	Jinxia Early Oil Flat Peach	Cultivars	Flat nectarine	China
119	Jinxia Oil Flat Peach	Cultivars	Flat nectarine	China
120	Ruipan 1	Cultivars	Flat peach	China
121	Ruipan 2	Cultivars	Flat peach	China
122	Ruipan 3	Cultivars	Flat peach	China
123	Tianjin Yellow Flesh	Landraces	Peach	China
124	Xi'an Apricot-flesh Peach	Landraces	Peach	China
125	Xizhuang 1	Landraces	Peach	China
126	Long 1-2-4	Landraces	Peach	China
127	Large Leaf Yellow Sweet Peach	Landraces	Peach	China
128	Nanshan Sweet Peach	Landraces	Peach	China
129	Nanshan Sweet Peach 1	Landraces	Peach	China
130	Green Leaf Winter Peach	Landraces	Peach	China
131	Red Leaf Winter Peach	Landraces	Peach	China
132	Lianyungang Winter Peach	Landraces	Peach	China
133	Fodu Winter Peach	Landraces	Peach	China
134	Peixian Winter Peach	Landraces	Peach	China
135	Zijinhong 3	Cultivars	Nectarine	China
136	Zijinhong 2	Cultivars	Nectarine	China
137	Zijinhong 1	Cultivars	Nectarine	China
138	Zaofengtian	Cultivars	Nectarine	China
139	Qidong Nectarine	Landraces	Nectarine	China
140	Kashi 1	Landraces	Peach	China

141	Shache Autumn Peach	Landraces	Peach	China
142	Xinjiang Yellow Flesh	Landraces	Peach	China
143	Xinjiang Large Sweet Kernel	Landraces	Peach	China
144	Black Peach	Landraces	Peach	China
145	Jinhua Black Peach 1	Landraces	Peach	China
146	Hairy Purple Peach 318	Landraces	Peach	China
147	Black Girl	Landraces	Peach	China
148	Purple Hairy Peach	Landraces	Peach	China
149	Rugao Purple Nectarine 2	Landraces	Nectarine	China
150	Rugao Purple Nectarine 3	Landraces	Nectarine	China
151	Purple Nectarine 5	Landraces	Nectarine	China
152	Purple Nectarine 8	Landraces	Nectarine	China
153	Purple Nectarine 9	Landraces	Nectarine	China
154	Yushuizhai Ornamental Peach	Landraces	Peach	China
155	Dazhao Yellow Peach	Landraces	Peach	China
156	Early Yellow Sweet Peach	Landraces	Peach	China
157	Yexian Yellow Peach 8	Landraces	Peach	China
158	Zhanghuang 3	Landraces	Peach	China
159	Nanjing White Sand	Landraces	Peach	China
160	Banhan	Landraces	Peach	China
161	Taqiao	Landraces	Peach	China
162	Liuhe Honey Peach	Landraces	Peach	China
163	Baimaoyuan	Landraces	Peach	China
164	Bingbaitao	Cultivars	Peach	Italy
165	Zhangbai 2	Landraces	Peach	China
166	Wujiang White	Landraces	Peach	China
167	Qingmaozi White Flower Peach	Landraces	Peach	China
168	Yanwohong	Landraces	Peach	China
169	Shiwo Honey Peach	Landraces	Peach	China
170	Weinan Sweet Peach	Landraces	Peach	China
171	Red Peach	Landraces	Peach	China
172	Yeji Red	Landraces	Peach	China
173	Beijing Yixianhong	Landraces	Peach	China
174	Shennong Red Flesh	Landraces	Peach	China
175	Summer Peach	Landraces	Peach	China
176	Shujihong	Landraces	Peach	China

177	Dahongpao	Landraces	Peach	China
178	Yixianbai	Landraces	Peach	China
179	Yixianhong	Landraces	Peach	China

Supplementary Table S2. Code and decoded levels of independent variables used in the RSM design.

Independent Variables	Symbols	Levels		
		-1	0	1
**Ultrasonic Time (min)**	X_1	35	40	45
**Ultrasonic Power (W)**	X_2	300	360	420
**Liquid-to-solid (mL/g)**	X_3	10/1	15/1	20/1
**Ultrasonic Temperature (°C)**	X_4	60	70	80

Supplementary Table S3. One-way ANOVA analysis

Factor	Sum of Squares	df	Mean Square	F	Significant
Methanol concentration (%)	0.203	6	0.034	4.194	0.013*
Ultrasonic time (min)	0.458	8	0.057	19.287	< 0.001**
Ultrasonic power (W)	0.037	5	0.007	8.362	0.001**
Liquid-to-solid ration (mL/g)	0.447	4	0.112	14.612	< 0.001**
Ultrasonic temperature (℃)	1.505	6	0.251	19.988	< 0.001**

Note:*indicates ($p < 0.05$), ** ($p < 0.01$).
